# Supplementary material for: Anchoring zero valence single atoms of nickel and iron on graphdiyne for hydrogen evolution
Source: Nat Commun. 2018 Apr 13;9:1460. doi: 10.1038/s41467-018-03896-4 (PMC5899097; doi:10.1038/s41467-018-03896-4)
Supplement: Supplementary file 1 — Supplementary Information [file 41467_2018_3896_MOESM1_ESM.pdf]

Supplementary Information for

Anchoring zero valence single-atom of nickel and iron on graphdiyne for hydrogen evolution

Yurui Xue[1], Bolong Huang[2], Yuanping Yi[1,3], Yuan Guo[1], Zicheng Zuo[1], Yongjun Li[1,3], Zhiyu Jia[1], Huibiao Liu[1,3] & Yuliang Li[1,3*]

[1]Key Laboratory of Organic Solids, Institute of Chemistry, the Chinese Academy of Sciences, Beijing 100190, PR China.

[2]Department of Applied Biology and Chemical Technology, The Hong Kong Polytechnic University, Hung Hom, Kowloon, Hong Kong SAR, China.

[3]University of Chinese Academy of Sciences, Beijing 100049, PR China.

Supplementary Figures

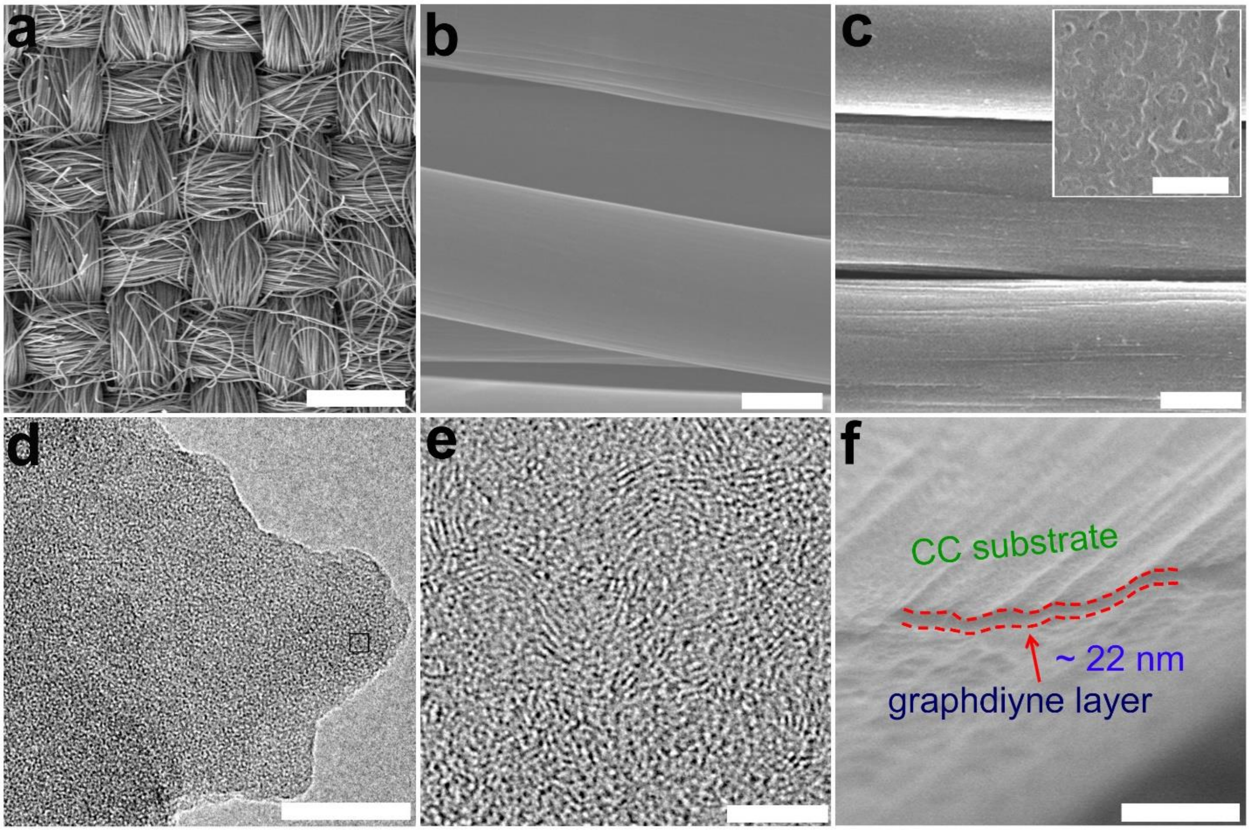

Supplementary Figure 1. Morphology characterizations

a Low- and **b** high-magnification SEM images of CC. Scale bars for a and b, respectively: 500 μm, 5 μm. **c** low- (Scale bar: 5 μm) and high-magnification (inset; scale bar: 300 nm) SEM images of GDF. **d** low- and **e** high-resolution TEM (HRTEM) images of GDF. Scale bars for **d** and **e**, respectively: 30 nm, 5 nm. **f** SEM image of GDF showing the layer thickness (~ 22 nm) measurements. Scale bar: 300 nm.

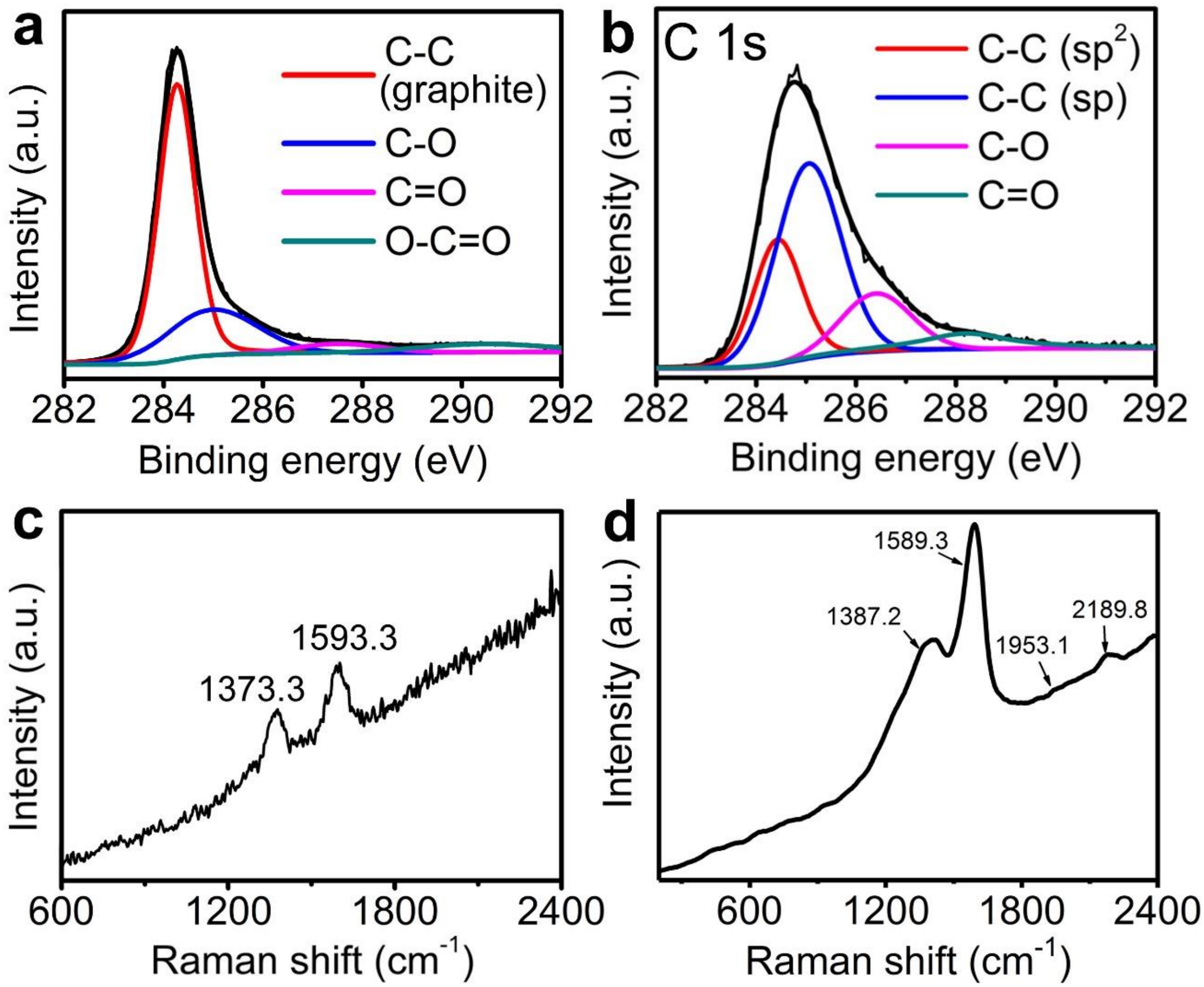

Supplementary Figure 2. XPS and Raman characterization of CC and GDF

High resolution XPS spectra of C 1s for **a** pure CC and **b** GDF; Raman spectra of **c** pure CC and **d** GDF.

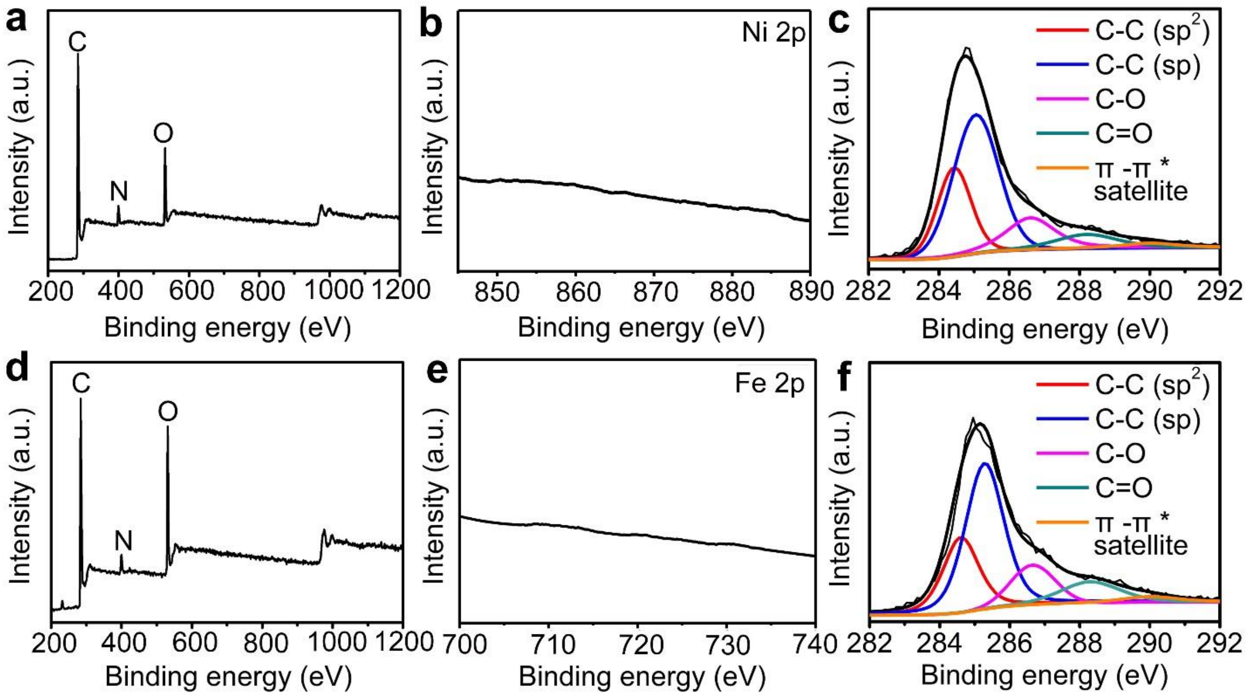

Supplementary Figure 3. XPS characterization of Ni/GD and Fe/GD

High resolution XPS spectra of **a** C 1s, **b** Ni 2p, and **c** XPS survey spectra of Ni/GD; High resolution XPS spectra of **d** C 1s, **e** Fe 2p, and **f** XPS survey spectra of Fe/GD.

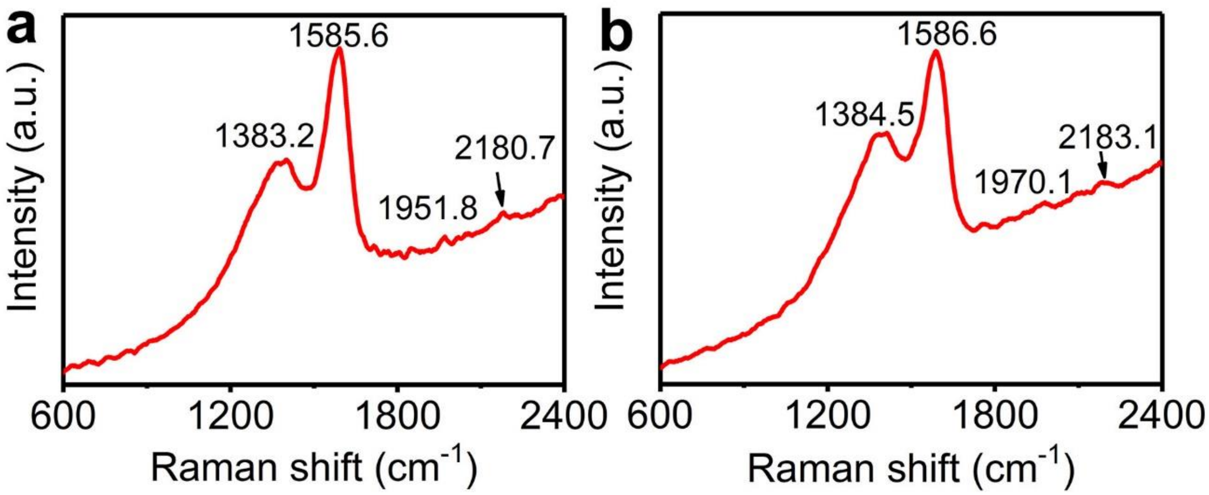

Supplementary Figure 4. Raman characterization of Ni/GD and Fe/GD

a Raman spectra of Ni/GD. **b** Raman spectra of Ni/GD. From Raman spectra, the diffraction peaks of the diyne groups of Ni/GD and Fe/GD shifted slightly, consistent with the formation of chemical bonds after TM atoms anchoring. The ratio of the D and G band intensities for both Ni/GD (0.87) and Fe/GD (0.85) are larger than that of GDF (0.77), suggesting more defects had formed, favoring the accessibility of more active sites and potentially increasing the catalytic efficiency.

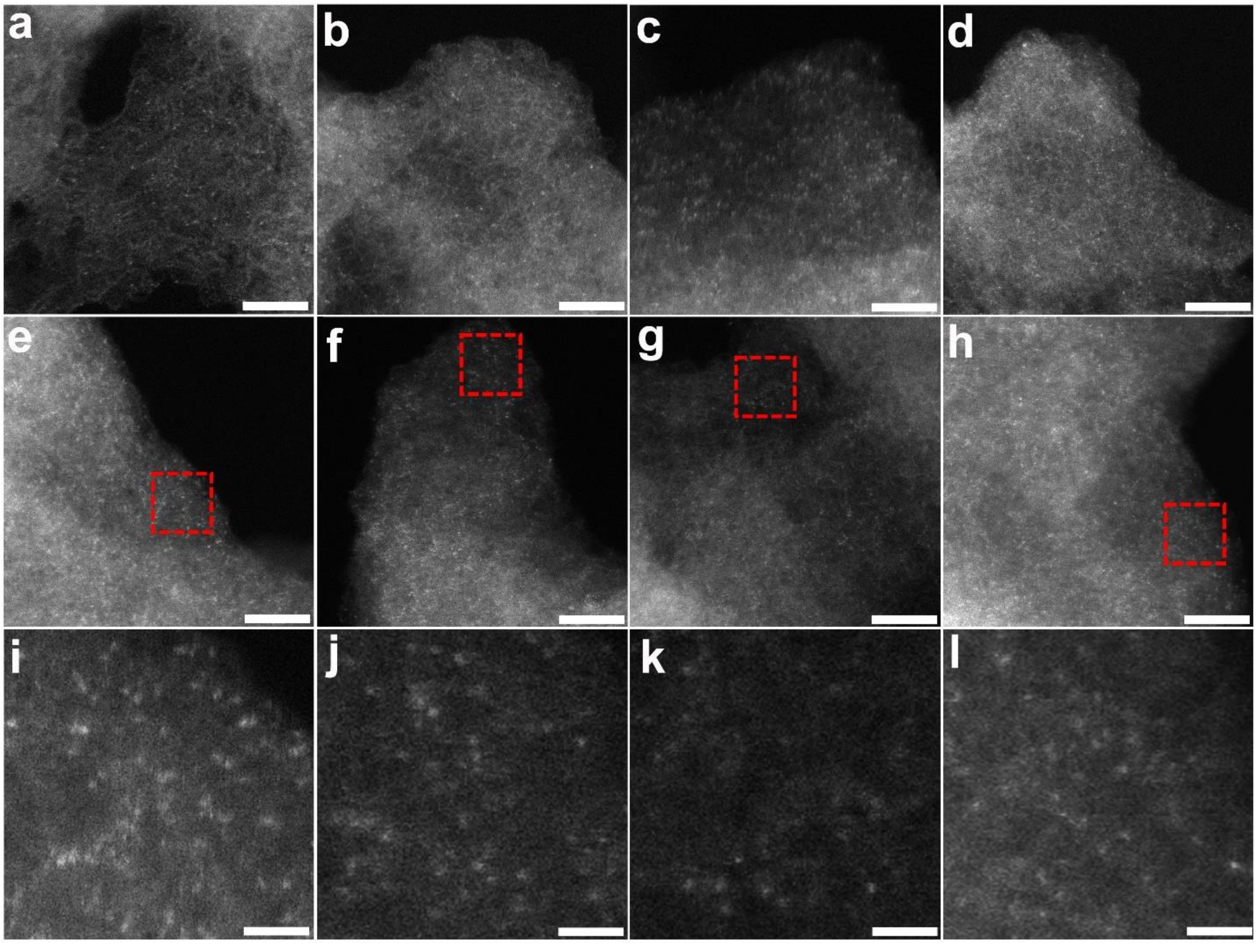

Supplementary Figure 5. Atomic resolution HAADF-STEM images of different areas of Ni/GD

a-h HAADF-STEM images taken from different regions of Ni/GD. Scale bars for **a**: 3 nm; scale bars for **b-h**: 5 nm. **i-l** high-magnification HAADF-STEM images taken from the marked area in **e-h**. Scale bars for **i-l**: 1 nm. White dots are single Ni atoms. Examination of different regions clearly showed that only Ni single atoms present in Ni/GD, further indicating the successful anchoring of Ni single atoms on GD.

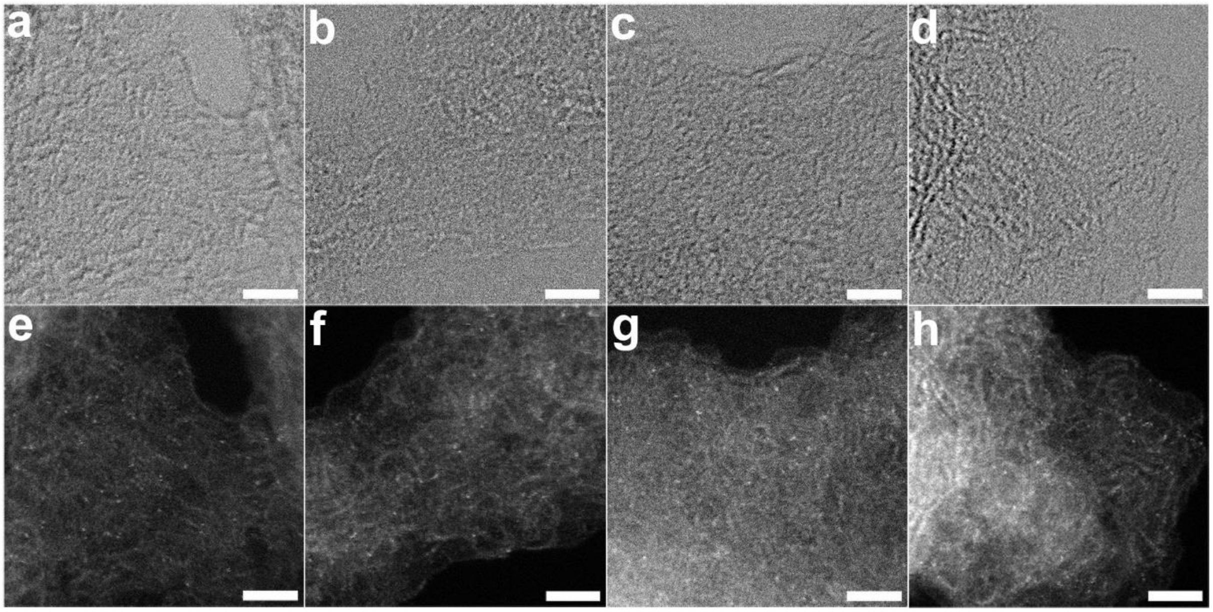

Supplementary Figure 6. Aberration-corrected scanning transmission electron microscopy (STEM) images of Ni/GD

a-d Higher-magnification TEM and **e-h** corresponding HAADF-STEM images recorded at different regions of Ni/GD ACs. Scale bars: 2 nm.

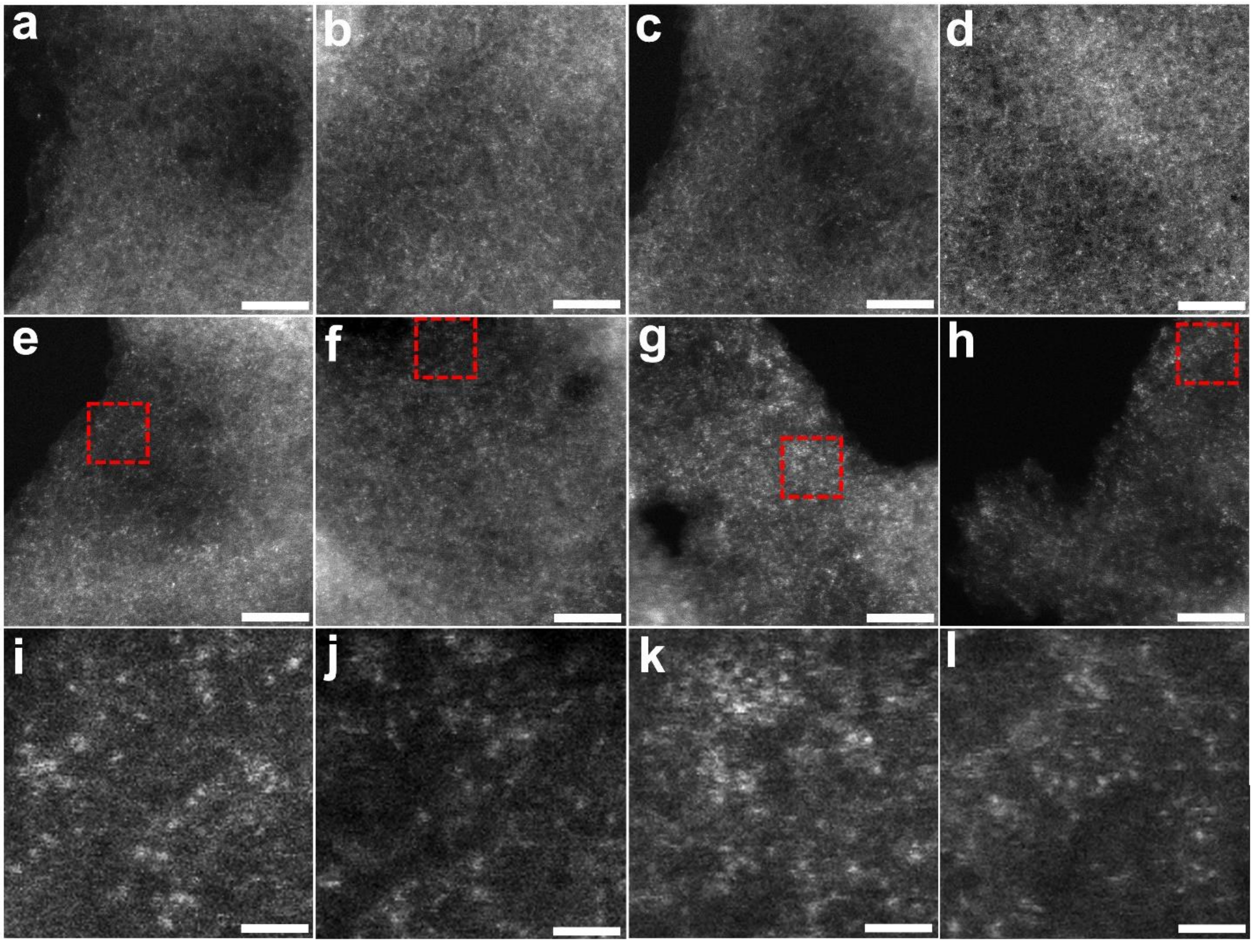

Supplementary Figure 7. Atomic resolution HAADF-STEM images of different areas of Fe/GD

a-h HAADF-STEM images taken from different regions of Fe/GD. Scale bars, 5 nm; **i-l** high-magnification HAADF-STEM images taken from the marked area in **e-h**. Scale bars for **i-l**: 1 nm. White dots are single Fe atoms. Examination of different regions clearly showed that only Fe single atoms present in Fe/GD, further indicating the successful anchoring of Fe single atoms on GD.

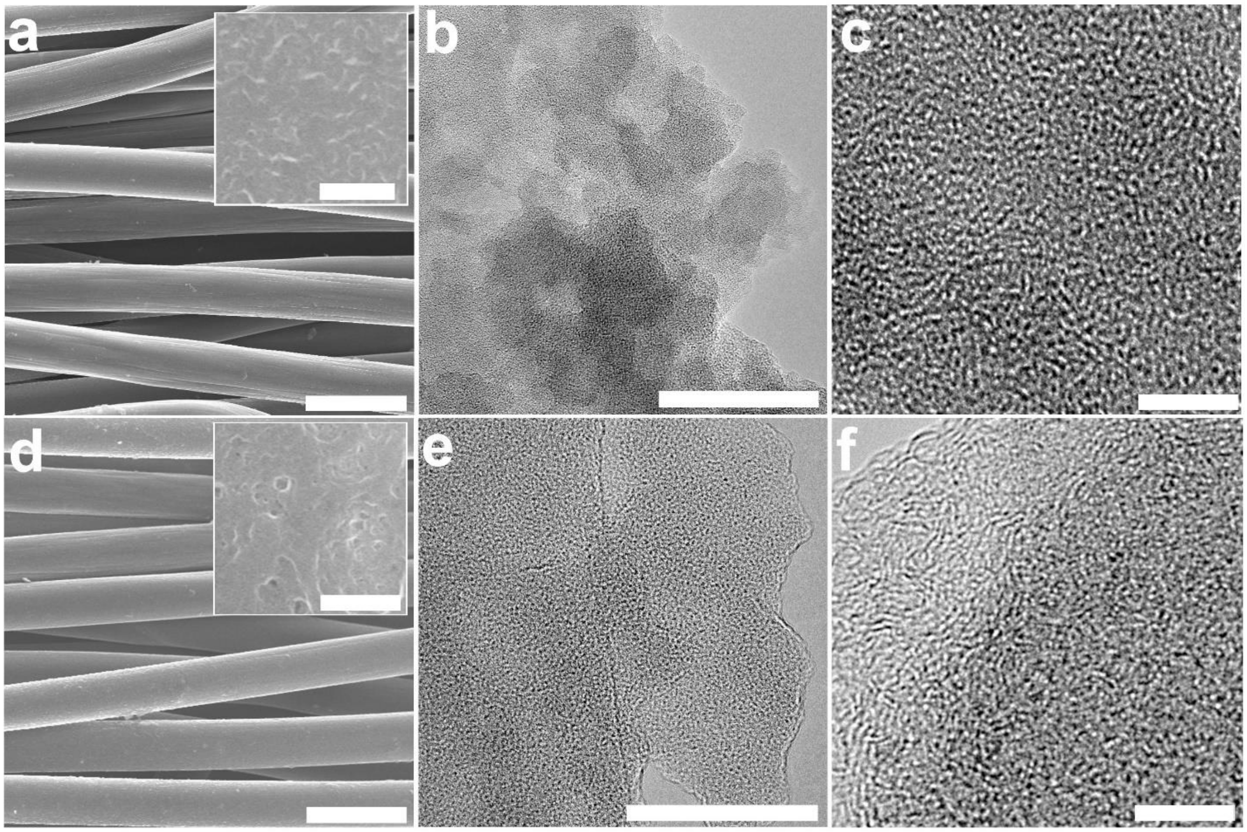

Supplementary Figure 8. Morphology characterizations

a Low- (scale bar, 20 μm) and high-magnification (inset; scale bar, 300 nm) SEM images of Ni/GD. **b** Low- (scale bar, 50 nm) and **c**, high-resolution (scale bar, 5 nm) TEM (HRTEM) images of Ni/GD. **d** Low- (sacle bar: 20 μm) and high-magnification (inset; scale bar: 300 nm) SEM images of Fe/GD. **e** Low- (scale bar, 50 nm) and **f** high-resolution (scale bar, 5 nm) TEM (HRTEM) images of Fe/GD.

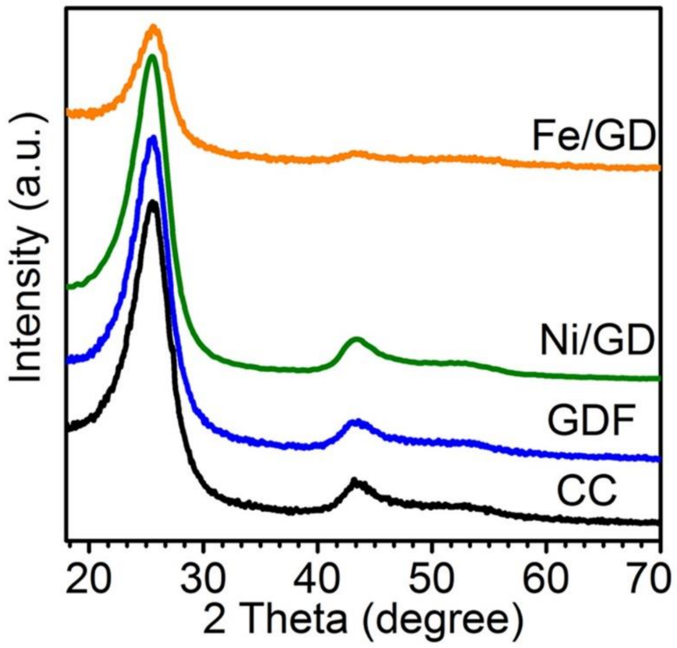

Supplementary Figure 9. XRD patterns of samples

XRD pattern of CC (black line), GDF (blue line), Ni/GD (green line) and Fe/GD (orange line). No characteristic patterns corresponding to Ni and Fe clusters/particles can be observed.

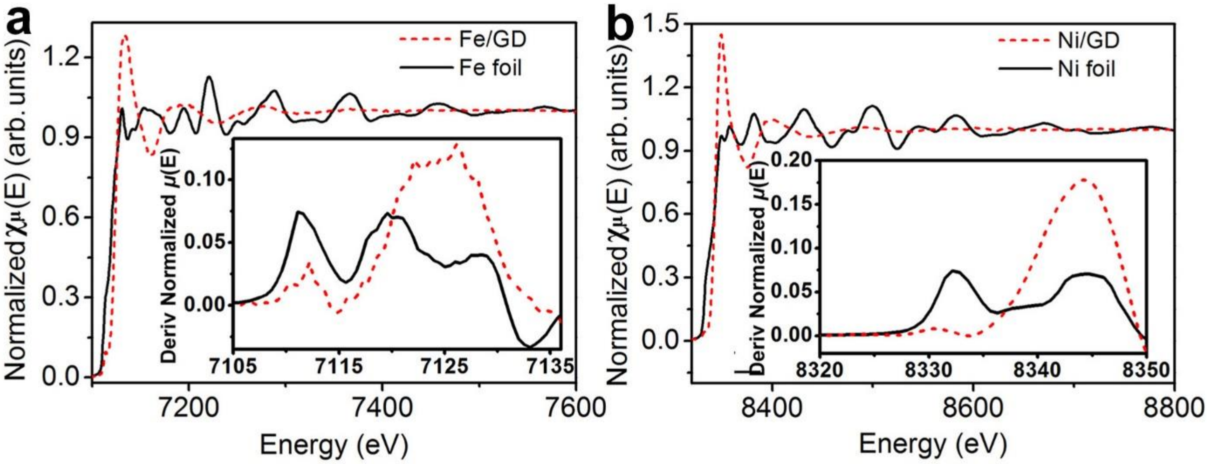

Supplementary Figure 10. Extended x-ray absorption fine structure (EXAFS) spectroscopy results of samples

a The normalized Fe K-edge XANES spectra and first derivative curves (inset) of different samples and references. **b** The normalized Ni K-edge XANES spectra and first derivative curves (inset) of different samples and references.

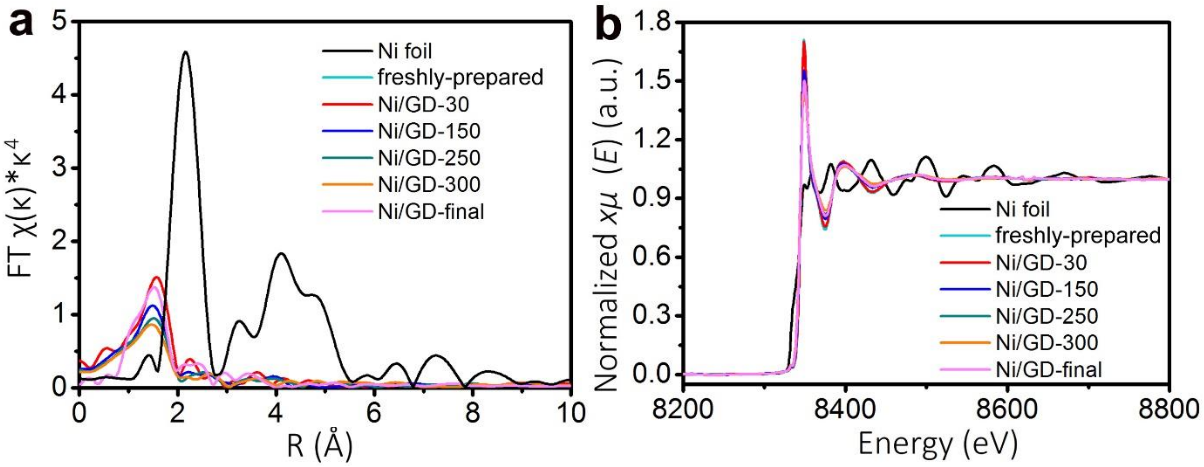

Supplementary Figure 11. XAS studies of Ni/GD catalysts at different temperature under 5% H_2/He

a *Ex situ* EXAFS spectra and **b** the normalized XANES spectra at the Ni K edge of Ni/GD at the Ni K-edge obtained at different reduction conditions. Ni foil was measured for comparison.

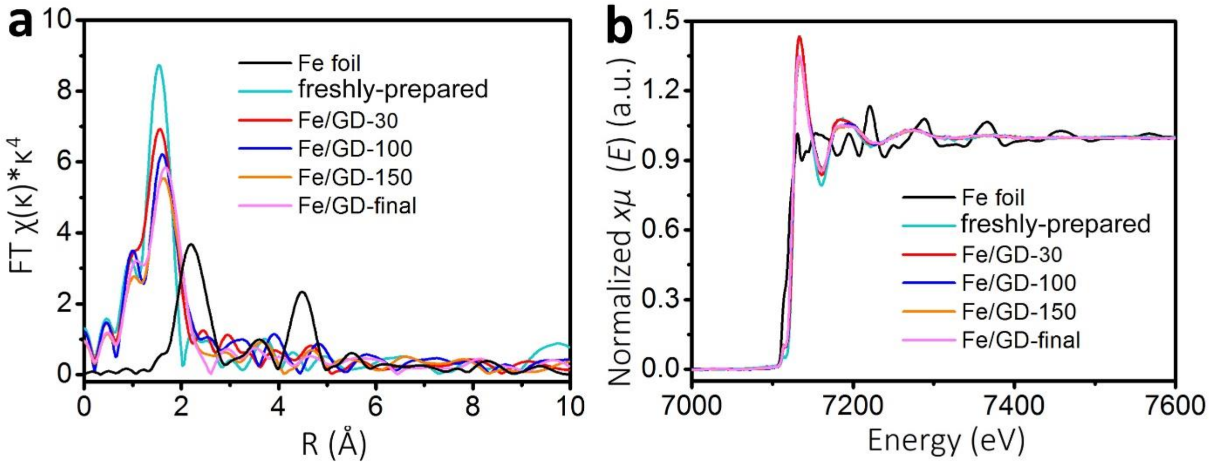

Supplementary Figure 12. XAS studies of Fe/GD catalysts at different temperature under 5% H_2/He

a *Ex situ* EXAFS spectra and **b** the normalized XANES spectra at the Fe K edge of Fe/GD at the Fe K-edge obtained at different reduction conditions. Fe foil was measured for comparison.

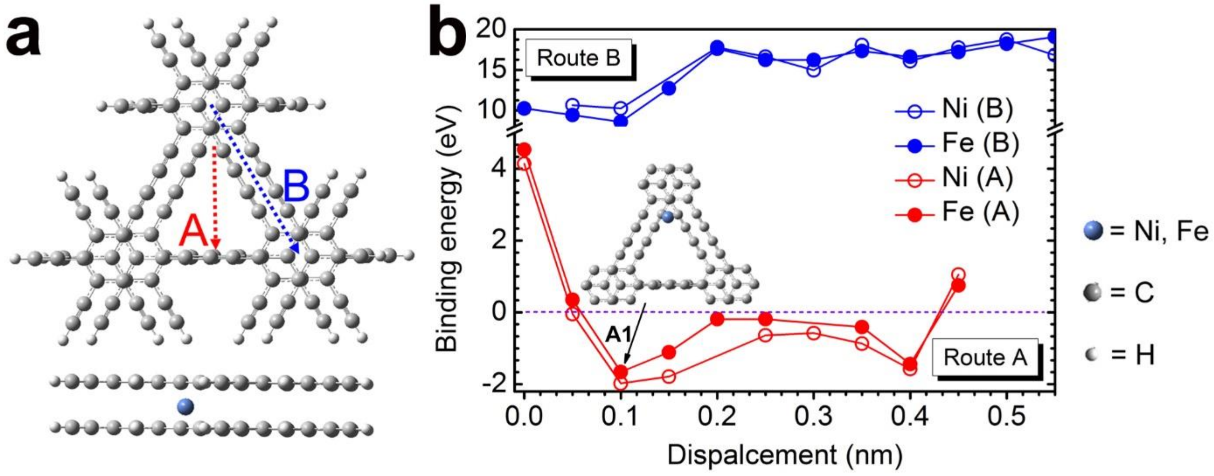

Supplementary Figure 13. Theoretical studies

a Possible adsorption sites for Ni/Fe atoms between GD layers along route A and route B (upper: top view; lower: side view). **b** Binding energies for Ni/Fe atom adsorption with bilayer GD as a function of displacement along the A and B directions in **a**. The AB stacking of the GD layers is accounted. We performed a potential energy surface scanning along two possible pathways (route A and route B) to calculate the binding energies between Ni/Fe atoms and two GD layers at the DFT-B3LYP/6-31G(d) level with BSSE corrected. Ni/Fe atoms are inclined to absorb along route A and the most favorable adsorption site is A2, giving the most negative binding energy (-1.98 and -1.66 eV for Fe and Ni, respectively).

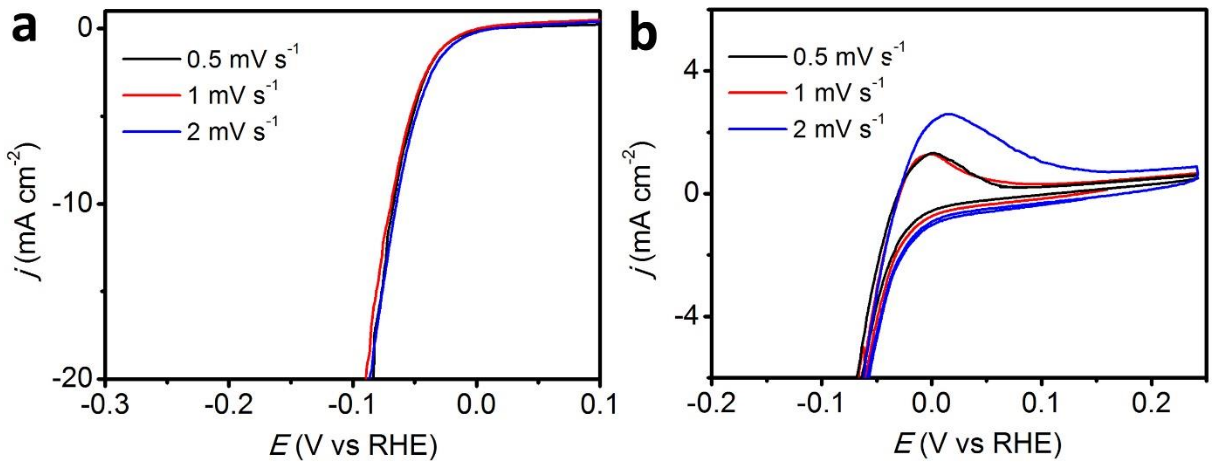

Supplementary Figure 14. Electrochemical measurements of Fe/GD in H_2-saturated 0.5 M H_2SO_4 electrolyte with various sweep rates

a Polarization curves of Fe/GD obtained at 0. 5 mV s^{-1}, 1 mV s^{-1} and 2 mV s^{-1}, respectively. **B** Electrochemical cyclic voltammogram curves of Fe/GD obtained at 0. 5 mV s^{-1}, 1 mV s^{-1} and 2 mV s^{-1}, respectively. It showed nearly the same current output with sweep rates. The shape and the size of the CV curves were almost the same at low sweep rates (0. 5 mV s^{-1}, 1 mV s^{-1}). After excluding the obvious effect of sweep rate on HER activity, we estimated the accurate overpotentials from polarization curve obtained at a slow sweep rate (0.5 mV s^{-1}) with negligible background current for further comparison with benchmarked electrocatalysts.

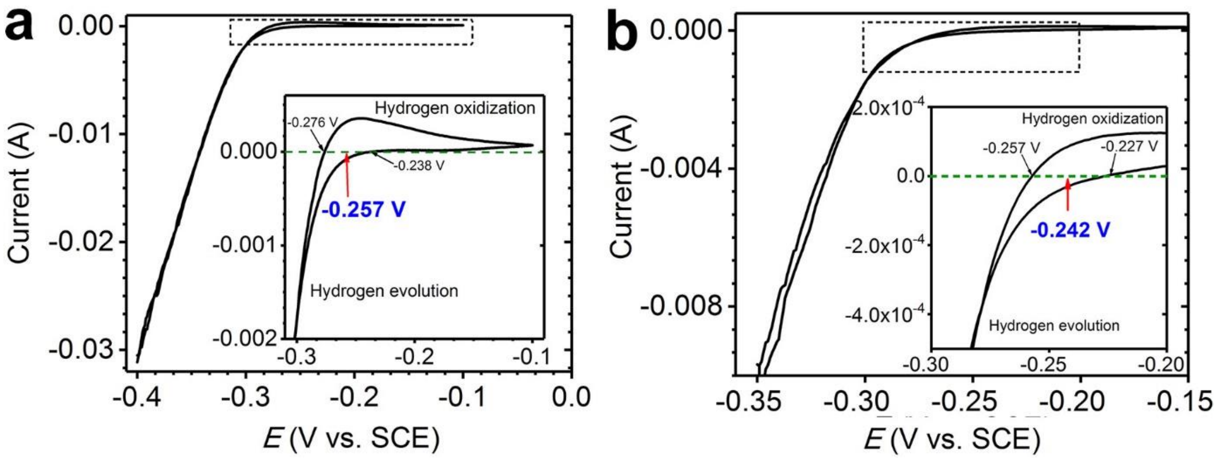

Supplementary Figure 15. Calibration of the saturated calomel electrode (SCE)

a This SCE was used in the durability tests of Ni/GD in 0.5 M H_2SO_4, E (RHE) = E (SCE) + 0.257 V. **b** This SCE was used in other electrochemical tests in 0.5 M H_2SO_4, E (RHE) = E (SCE) + 0.242 V. In all measurements, SCE was calibrated with respect to RHE. The calibration was performed in the high purity hydrogen saturated electrolyte with a Pt foil as the working electrode. Cyclic voltammetry (CV) was run at a scan rate of 1 mV s^{-1}, and the average of the two potentials at which the current crossed zero was taken to be the thermodynamic potential for the hydrogen electrode reaction.

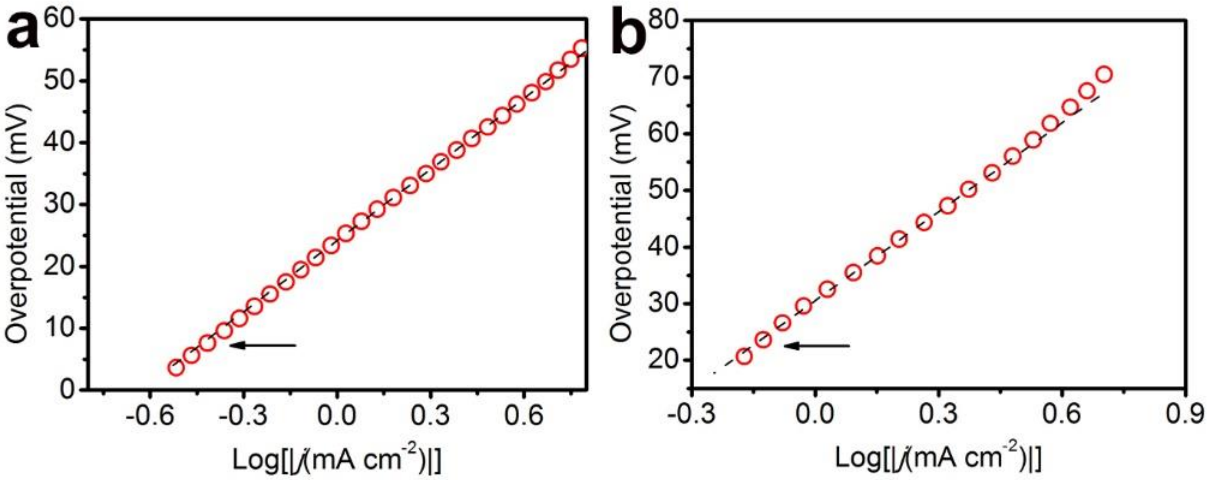

Supplementary Figure 16. Determination of the onset overpotentials of samples

Tafel plot in the region of low current densities of **a** Fe/GD and **b** Ni/GD in 0.5 M H_2SO_4. The onset overpotential is determined by the potential when the plot starts to deviate from the linear region as indicated by the arrow.

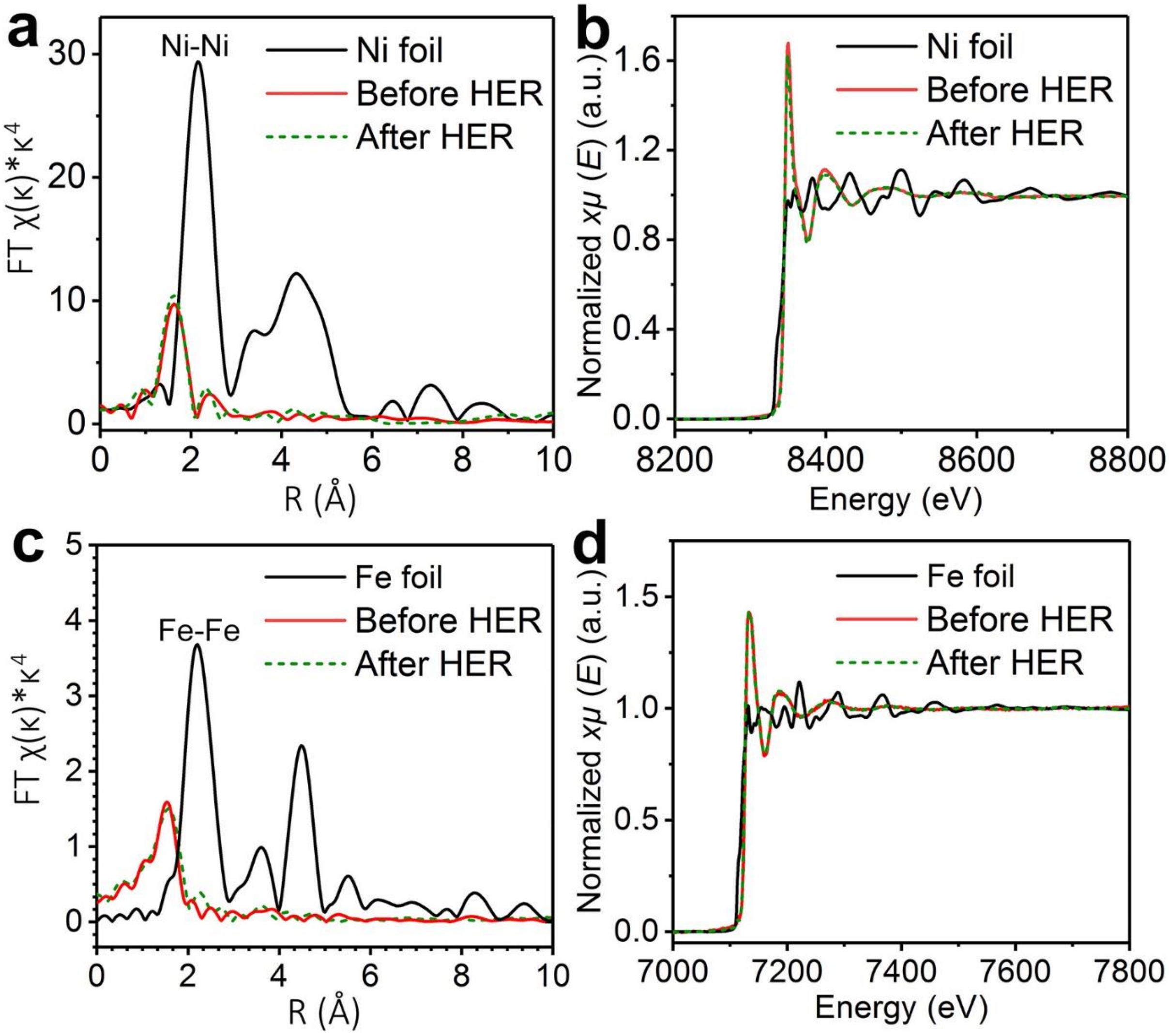

Supplementary Figure 17. XAS studies of samples obtained before and after HER measurements

a *Ex situ* EXAFS and **b** the normalized XANES spectra at the Ni K edge of Ni/GD obtained before (red line) and after (green dashed line) HER measurements. **c** *Ex situ* EXAFS and **d** the normalized XANES spectra at the Fe K edge of Fe/GD obtained before (red line) and after (green dashed line) HER measurements.

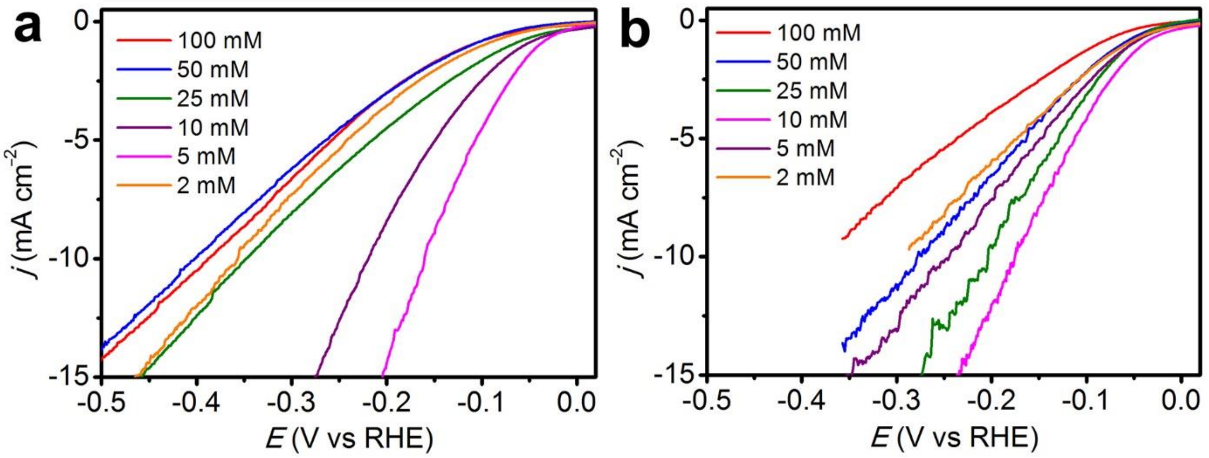

Supplementary Figure 18. Effects of the metal ion concentrations on the catalytic activities

Polarization curves (no iR-correction) of **a** Ni-GD and **b** Fe-GD obtained at different ion concentrations at a fixed deposition time of 150 s.

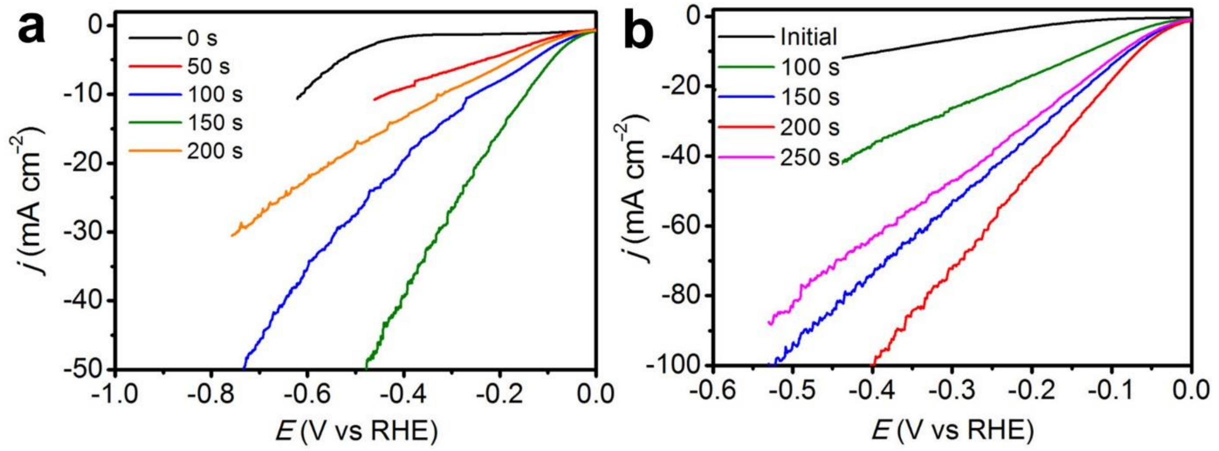

Supplementary Figure 19. Effects of the deposition time on the catalytic activities

Polarization curves (no iR-correction) of **a** Ni-GD and **b** Fe-GD obtained at different deposition times. As expected, with the increasing of the deposition time, the HER catalytic activities increased, and reached the best performances for both Ni-GD and Fe-GD. However, a longer deposition time will lead to the decrease of their catalytic performances.

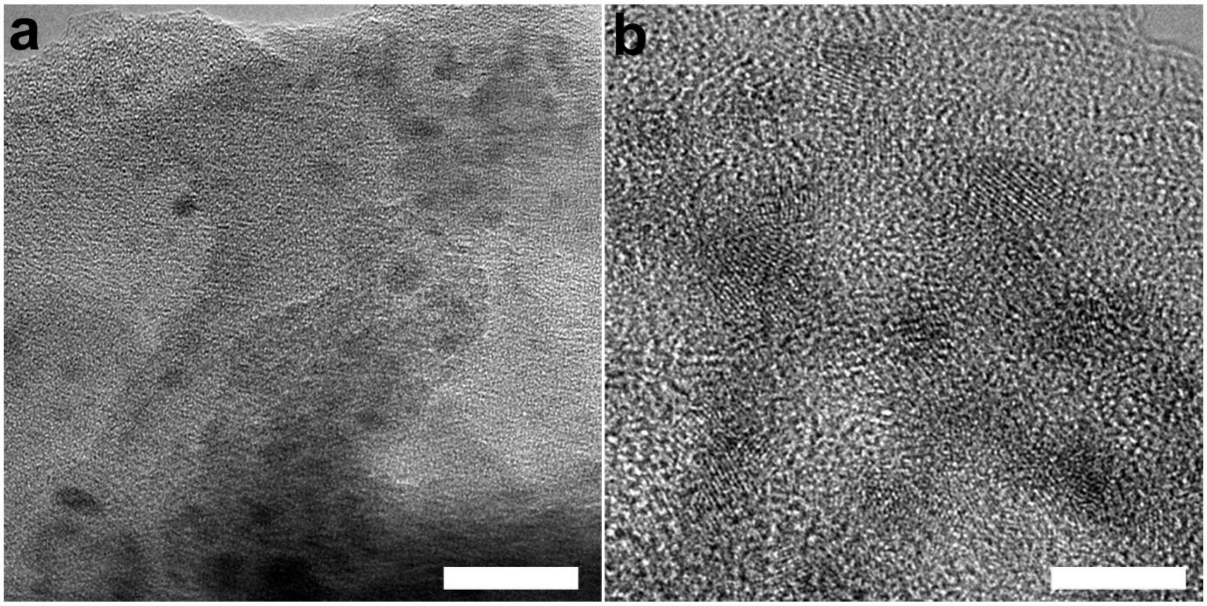

Supplementary Figure 20. Characterization of the Fe nanoparticles decorated GD (Fe NPs-GD)

a TEM (scale bar, 20 nm) and **b** high-resolution TEM (scale bar, 5 nm) images of Fe nanoparticles decorated GD (Fe NPs-GD).

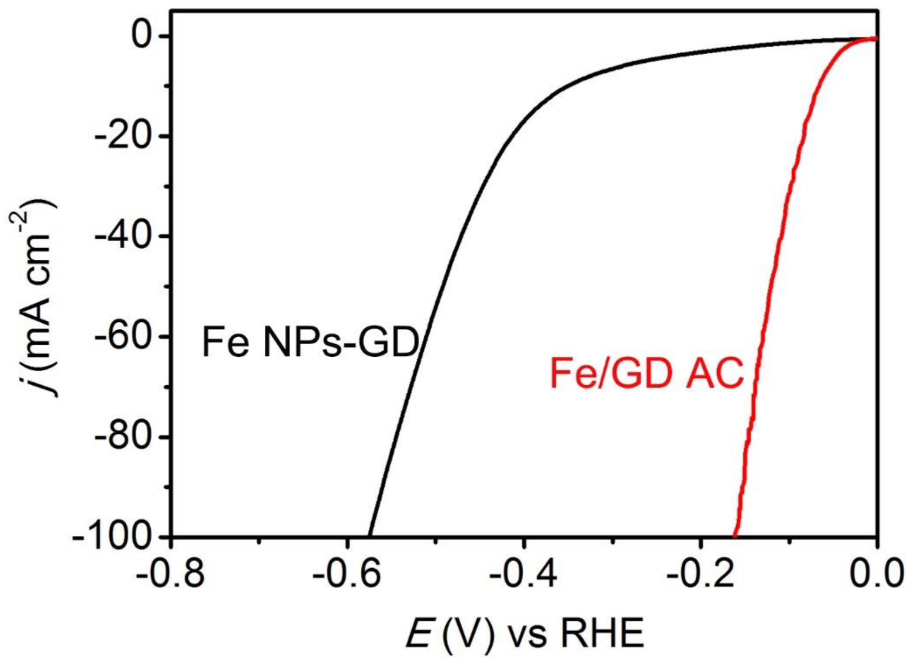

Supplementary Figure 21. Comparison of the HER activities of Fe nanoparticles decorated GD (Fe NPs-GD) and Fe/GD

Polarization curves of Fe NPs-GD (black line) and Fe/GD ACs (red line).

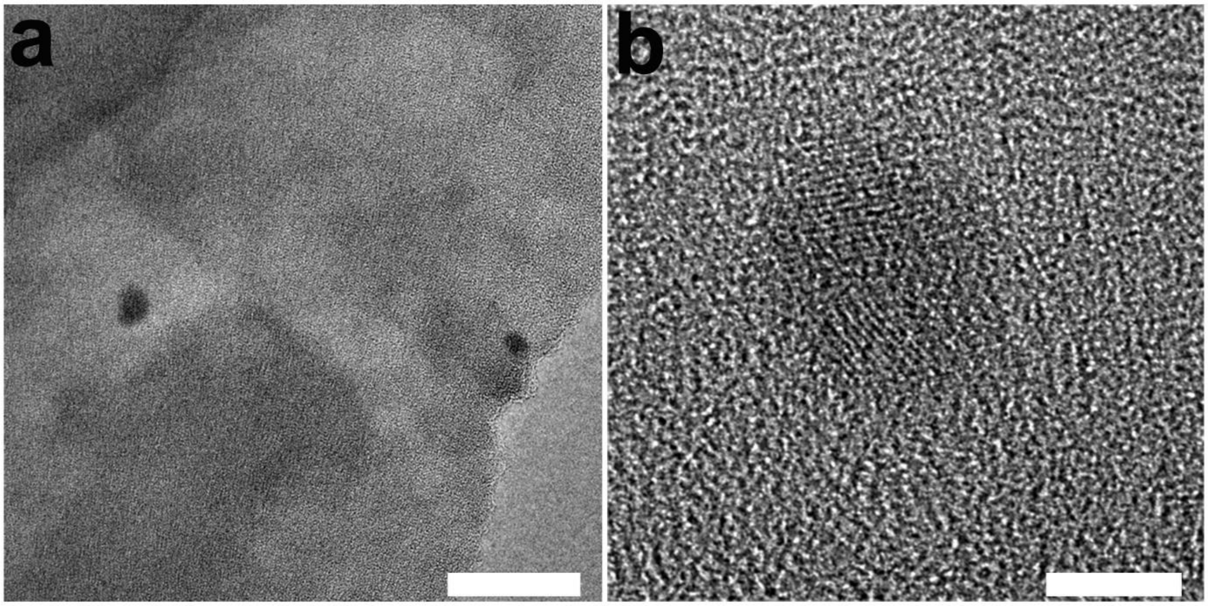

Supplementary Figure 22. Characterization of the Ni nanoparticles decorated GD (Ni NPs-GD)

a TEM (scale bar, 50 nm) and **b** high-resolution TEM (scale bar, 5 nm) images of Ni nanoparticles decorated GD (Ni NPs-GD).

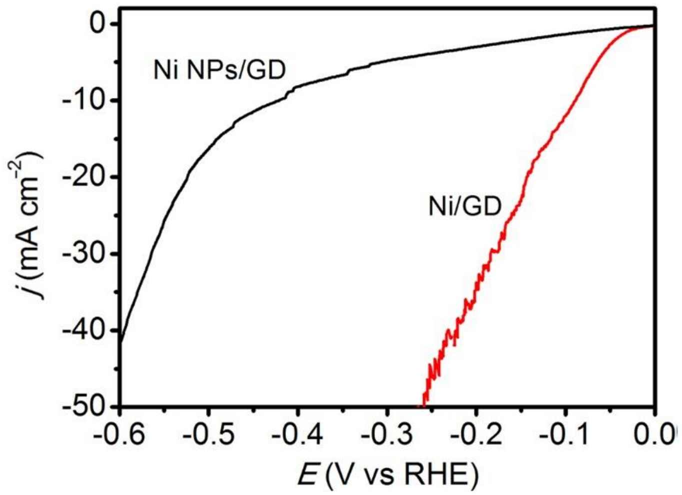

Supplementary Figure 23. Comparison of the HER activities of Ni nanoparticles decorated GD (Ni NPs-GD) and Ni/GD

Polarization curves of Ni NPs-GD (black line) and Ni/GD ACs (red line).

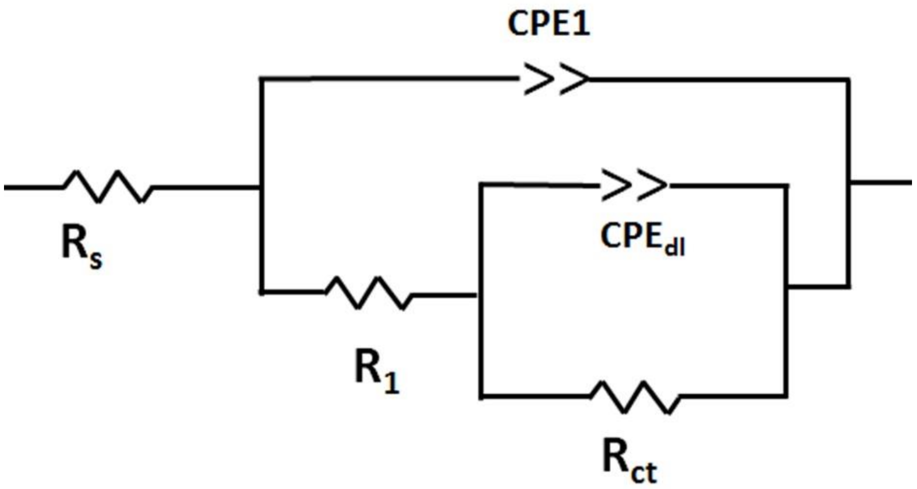

Supplementary Figure 24. The simplified equivalent circuit model

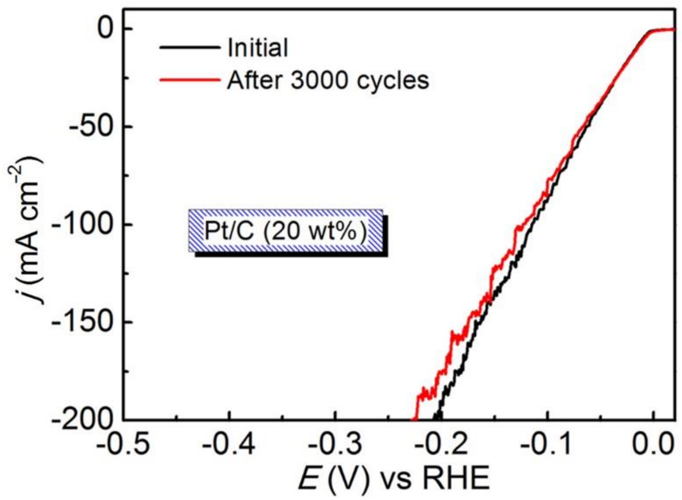

Supplementary Figure 25. Stability test of Pt/C (20 wt%)

Polarization curves of Pt/C (20 wt%) obtained before (black line) and after (red line) 3000 cycling tests.

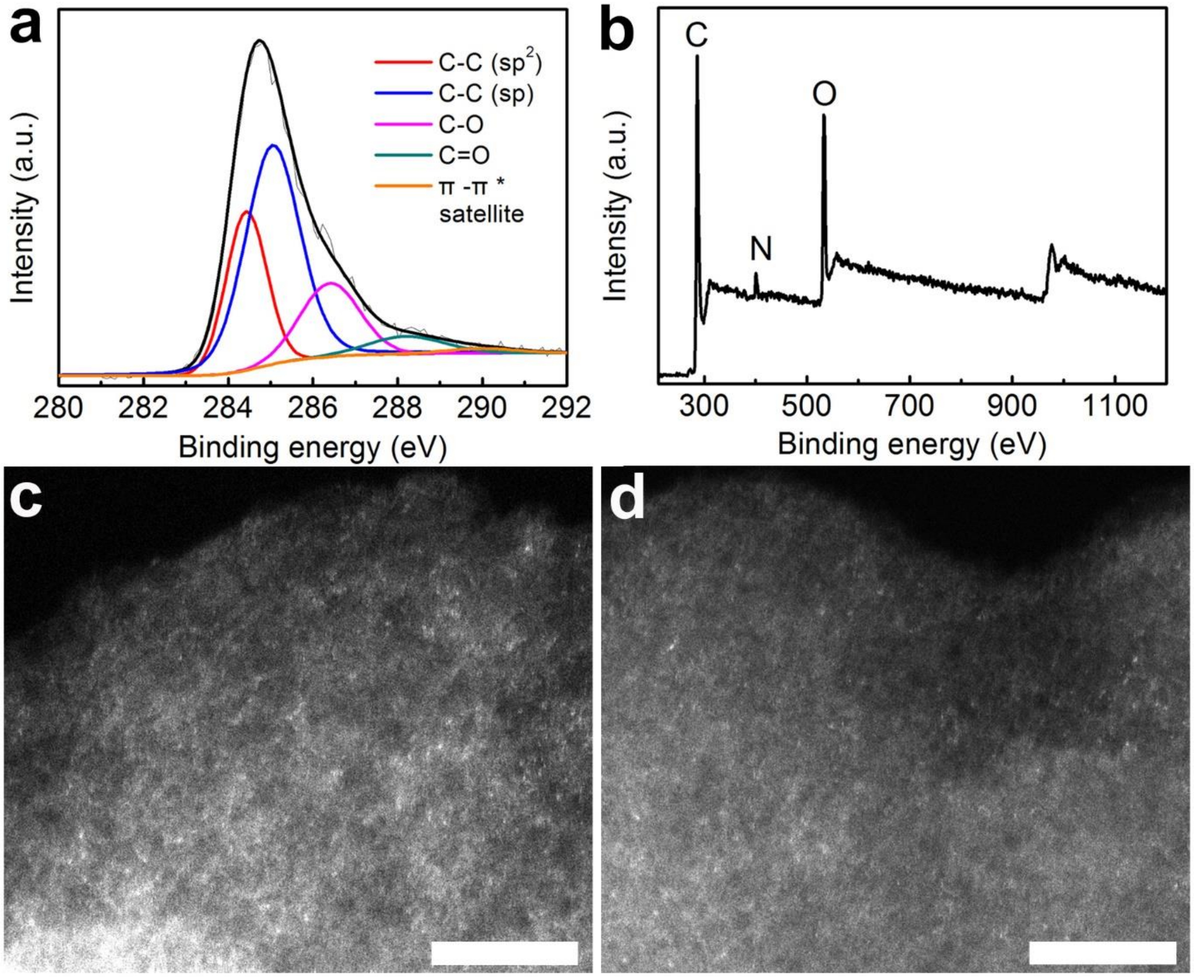

Supplementary Figure 26. Characterization of Ni/GD after cycling tests

a High resolution C 1s and **b** XPS survey spectra of Ni/GD. The C 1s XPS peak for Ni/GD can be deconvoluted into five sub-peaks located at 284.4, 285.1, 286.6, 288.2, and 290.0 eV, corresponding to the C 1s orbital of C–C (sp^2), C–C (sp), C–O, C=O, and the π-π* shake-up satellite peak, respectively. The area ratio of the sp– and sp^2– hybridized carbon atoms was 2, revealing the integrity of GD structure. **c**, **d** HAADF-STEM images taken from different regions of Ni/GD after 5000 cycling tests. Scale bars, 5 nm. No Ni clusters or particles can be observed from the HAADF images, indicating that the Ni atoms are firmly fixed on graphdiyne.

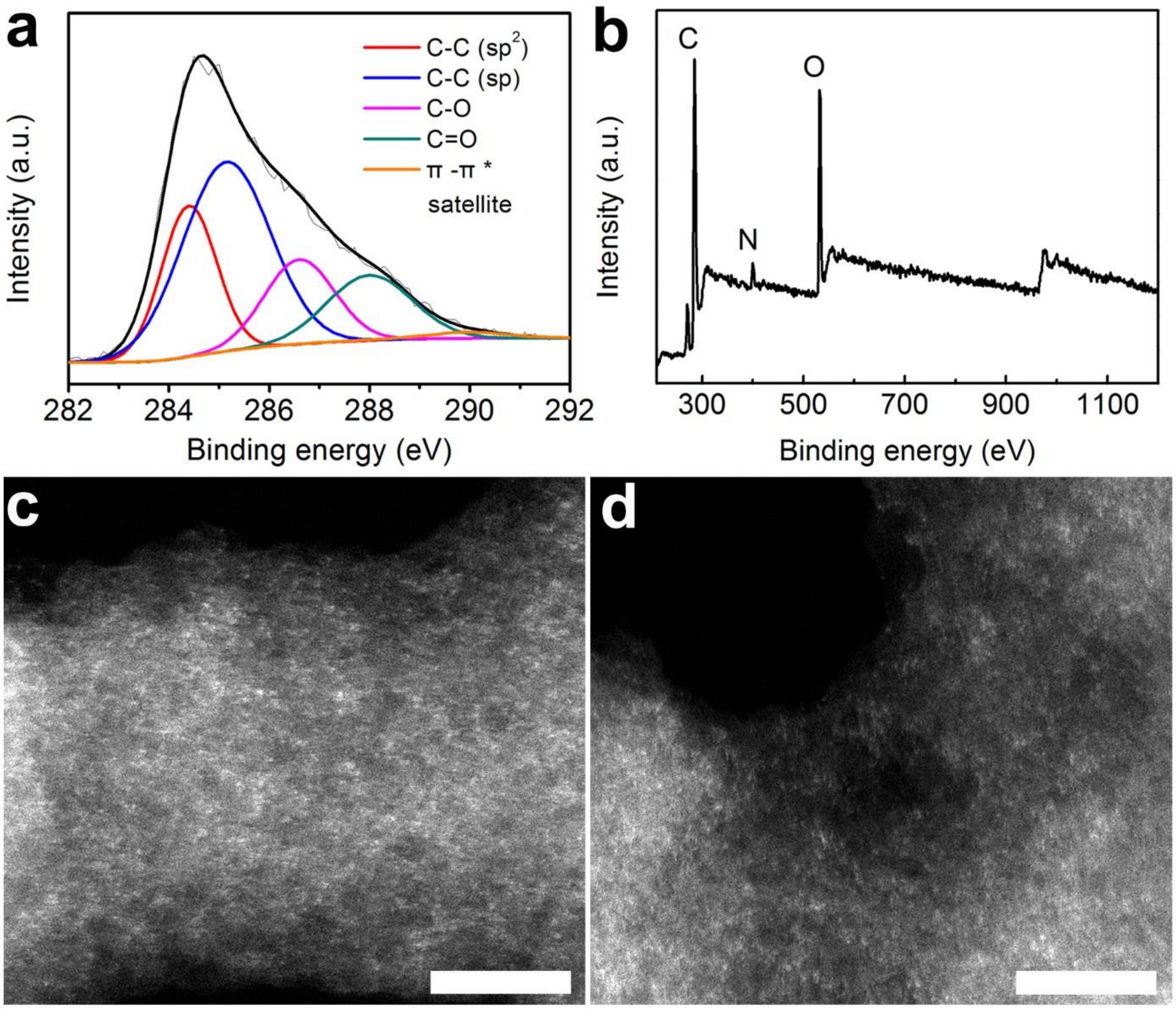

Supplementary Figure 27. Characterization of Fe/GD after cycling tests

a High resolution C 1s and **b** XPS survey spectra of Fe/GD. The C 1s XPS peak for Fe/GD can be deconvoluted into five sub-peaks located at 284.4, 285.1, 286.6, 288.2, and 290.0 eV, corresponding to the C 1s orbital of C–C (sp^2), C–C (sp), C–O, C=O, and the π-π* shake-up satellite peak, respectively. The area ratio of the sp– and sp^2– hybridized carbon atoms was 2, revealing the integrity of GD structure. **c, d** HAADF-STEM images taken from different regions of Fe/GD after 5000 cycling tests. Scale bars, 5 nm. No Fe clusters or particles can be observed from the HAADF images, indicating that the Fe atoms are firmly fixed on graphdiyne.

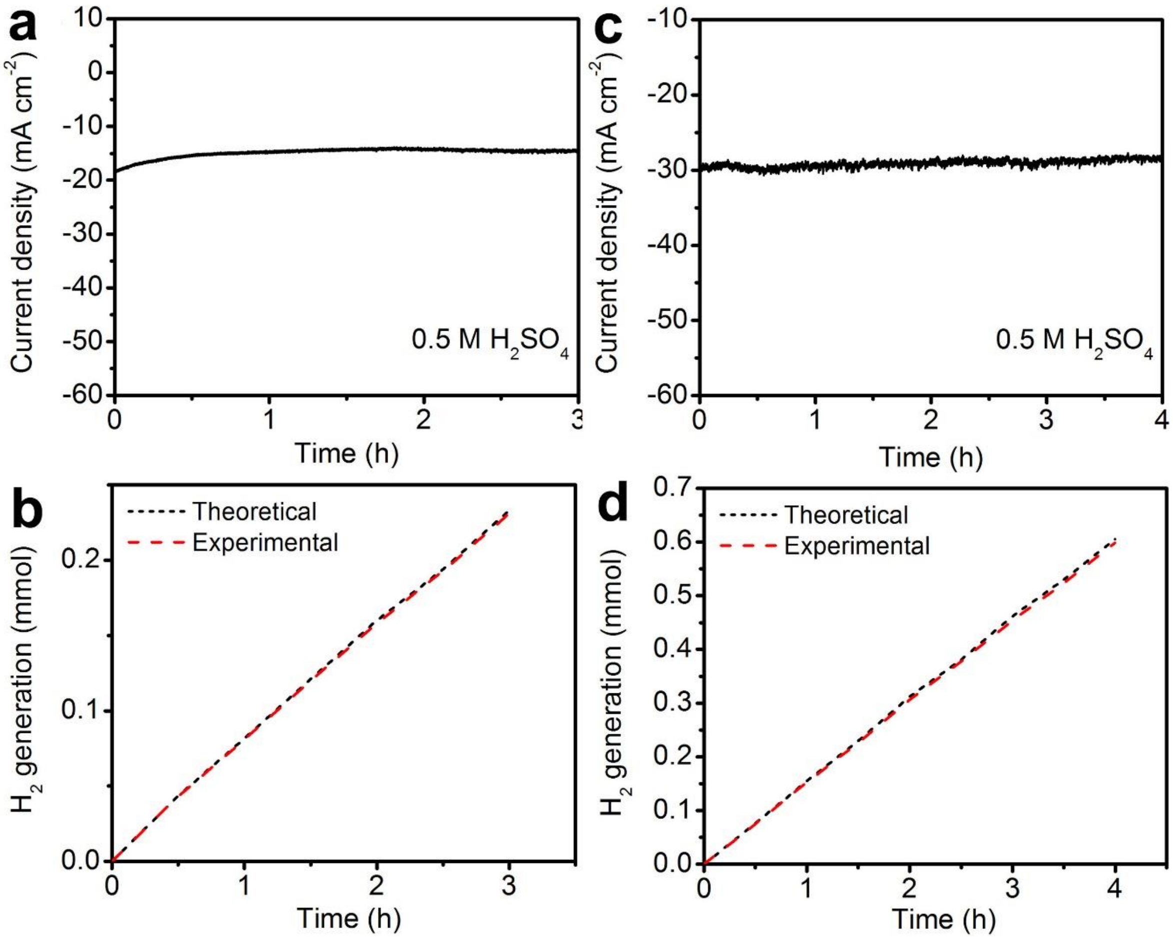

Supplementary Figure 28. Faraday efficiencies of catalysts

a Current versus time during the controlled potential (-0.1 V vs RHE) electrolysis of Ni/GD. **b** The real H_2 volume and theoretical volume (black) versus time gave a Faraday efficiency higher than 98 % (calculate on the data of 3 h). **c** Current versus time during the controlled potential (-0.01 V vs RHE) electrolysis of Fe/GD. **d** The real H_2 volume and theoretical volume (black) versus time gave a Faraday efficiency higher than 98 % (calculate on the data of 4 h).

Supplementary Tables

Supplementary Table 1. Fitting parameters of Ni and Fe K-edge EXAFS curves for Ni/GD and Fe/GD, respectively

Sample	Shell	CN [a]	R (Å) [b]	σ^2 (Å^2·10^3) [c]	ΔE0 (eV) [d]	R factor (%)
Ni/GD	Ni-C	12.0	2.05	6.1	9.89	0.26
Fe/GD	Fe-C	12.0	2.03	5.1	-8.7	0.93

[a] *N*: coordination numbers; [b] *R*: bond distance; [c] σ^2: Debye-Waller factors; [d] ΔE_0: the inner potential correction. *R* factor: goodness of fit. S_0^2, 0.912, was obtained from the experimental EXAFS fit of NiO reference by fixing CN as the known crystallographic value and was fixed to all the samples.

Supplementary Table 2. Binding energies, charge transfer amounts, and bond lengths between TM atoms and the nearest carbon atoms

Binding sites	TM atoms	Binding energy (eV) [a]	Charge transfer (e)	Bond length (Å)
S1	Ni	-3.72	0.127	1.93
	Fe	-1.22	0.587	1.80
S2	Ni	-1.39	0.149	2.10
	Fe	-0.37	0.674	1.98

[a] The binding energies were calculated with the basis set superposition error (BSSE) corrected.

Supplementary Table 3. Comparison of overpotential (η) at current density of 10 mA cm^{-2} and Tafel slopes of Ni/GD and Fe/GD with recently reported catalysts in 0.5 M H_2SO_4 aqueous solution

Catalyst	η at j = 10 mA cm^{-2} (mV)	Tafel slope (mV dec^{-1})	Ref.
Ni/GD	86	45.8	This work
Fe/GD	66	37.8	This work
Ni-doped graphene	180	45	*Angew. Chem. Int. Ed.* **54**, 14031–14035 (2015)
Co-NG	147	82	*Nat. Common.* **6**, 8668 (2015)
Ni-Mo-S nanosheet	200	85.3	*Sci. Adv.* **1**, e1500259 (2015)
Pt NWs/SL-Ni(OH)$_2$	95	--	*Nat. Common.* **6**, 6430 (2015)
MoC$_x$ nano-octahedra	142	53	*Nat. Common.* **6**, 6512 (2015)
Edge-terminated MoS$_2$	149	49	*Nat. Common.* **6**, 7493 (2015)
Mesoporoous MoS$_2$	233	50	*Nat. Mater.* **11**, 963–969 (2012)
SV MoS$_2$	170	60	*Nat. Mater.* **15**, 48–53 (2016)
CoMoS$_x$	207 (at 5 mA cm^{-2})	--	*Nat. Mater.* **15**, 197–203 (2016)
CoN$_x$/C	133	57	*Nat. Common.* **6**, 7992 (2015)
CoNi@NC	142	104	*Angew. Chem. Int. Ed.* **54**, 2100–2104 (2015)
Co-NRCNT	260	69	*Angew. Chem. Int. Ed.* **53**, 4372–4376 (2014)
CoS$_2$/RGO	278	82	*Angew. Chem. Int. Ed.* **53**, 12594–12599 (2014)
CoS$_2$/RGO-CNT	142	51	
FeCo@N-graphene (S600)	262	74	*Energy Environ. Sci.* **8**, 3563–3571 (2015)
Exfoliated WS$_2$	210	60	*Nat. Mater.* **12**, 850–855 (2013)
WS$_2$@P,N,O-graphene	125	52.7	*Adv. Mater.* **27**, 4234–4241 (2015)

(GO 8 wt%)Cu–MOF/GC	~170	84	*Adv. Funct. Mater.* **23**, 5363–5372 (2013)
Cu_2MoS_4/C	~320	95	*Energy Environ. Sci.* **5**, 8912–8916 (2012).
MPSA/GO-1000	163	89	*Angew. Chem. Int. Ed.* **55**, 2230–2234 (2016).
C_3N_4@NG hybrid	240	51.5	*Nat. Commun.* **5**, 3783 (2014)
g-C_3N_4 nanoribbon-G	207	54	*Angew. Chem. Int. Ed.* **53**, 13934–13939 (2014)

Supplementary Table 4. Comparison of the onset η and exchange current density (j_0) of Ni/GD and Fe/GD with recently reported catalysts in 0.5 M H_2SO_4 aqueous solution

Catalysts	Onset η (mV)	j_0 (A cm^{-2})	Ref.
Fe/GD	9	2.9×10^{-4}	This work
Ni/GD	23	2.5×10^{-4}	This work
Ni--doped graphene	50	5.3×10^{-5}	*Angew. Chem. Int. Ed.* **54**, 14031–14035 (2015)
Co-N-doped graphene	30	1.25×10^{-4}	*Nat. Common.* **6**, 8668 (2015)
SV MoS_2	46	1.0×10^{-6} – 1.3×10^{-5}*	*Nat. Mater.* **15**, 48–53 (2016)
CoP		1.4×10^{-4}	*Angew. Chem. Int. Ed.* **53**, 5427-5430 (2014)
CoPS		5.6×10^{-5}	*Nat. Mater.* **14**, 1245–1251 (2015)
2T WS_2	80	2.0×10^{-5}	*Nat. Mater.* **12**, 850–855 (2013)
Hydrazine-treated MoS_2 particles	200	1.7×10^{-5}	*Nat. Commun.* **7**, 11857 (2016)
Hydrazine-treated MoO_x/MoS_2 nanowires	200	7.5×10^{-6}	*Nat. Commun.* **7**, 11857 (2016)
Ni-Co based	65		*Adv. Energy Mater.* **5**, 1402031 (2015)
Ni_5P_4	140		*Angew. Chem. Int. Ed.* **54**, 12361–12365 (2015)
$Ni/NiO/CoSe_2$	30	1.4×10^{-5}	*Angew. Chem. Int. Ed.* **52**, 8546 – 8550 (2013)
$NiSe_2$	117	4.7×10^{-6}	*Angew. Chem. Int. Ed.* **55**, 6919 – 6924 (2016)
Ni_2P/Ti		3.3×10^{-6}	*J. Am. Chem. Soc.* **135**, 9267–9270 (2013)
Ni-Mo	65	8.4×10^{-7}	*ACS Nano* **10**, 10397–10403 (2016)
Ni_3S_2	91.6	4.92×10^{-4}	*Nano Energy* **36**, 85–94 (2017)
$Ni_{12}P_5$	143 ± 3		*ACS Nano* **8**, 8121–8129 (2014)
Fe_2P		9.6×10^{-5}	*Nano Energy* **12**, 666–674 (2015)

$Fe_{1-x}Co_xS_2$/CNT	120		*J. Am. Chem. Soc.* **137**, 1587–1592 (2015)
$CoSe_2$	174	6.38×10^{-8}	*J. Am. Chem. Soc.* **138**, 5087–5092 (2016)
Co-C-N	212		*J. Am. Chem. Soc.* **137**, 15070–15073 (2015)
Co_2P	167		*Nano Energy* **9**, 373–382 (2014)
CoS_2/RGO-CNT		6.26×10^{-5}	*Angew. Chem. Int. Ed.* **53***,* 12594–12599 (2014)
CoN_x/C		9.6×10^{-5}	*Nat. Commun.***6***,* 7992 (2015)
Mo_2C@NC	60	9.6×10^{-5}	*Angew. Chem. Int. Ed.* **54**, 10752–10757 (2015)
MoSSe@rGO	135		*Nano Energy* **29**, 46–53 (2016)
MoS_x	146		*Nano Energy* **11**, 11–18 (2015)
MoS_2	120		*J. Am. Chem. Soc.* **137**, 7365–7370 (2015)

Supplementary Table 5. Comparison of the TOF values of Ni/GD with other reported catalysts in 0.5 M H_2SO_4 aqueous solution

Catalysts	TOF at η = 100 mV	Reference
Ni/GD	1.59	This work
Fe/GD	4.15	This work
MoS_2-edge	~0.1	*Science* **317**, 100–102 (2007).
Co-NG	0.101	*Nat. Commun.* **6**, 8668 (2015)
CoN_x/C	0.39	*Nat. Common.* **6**, 7992 (2015)
Ni-C-N NSs	0.44	*J. Am. Chem. Soc.* **138**, 14546–14549 (2016)
Ni_2P	0.015	*J. Am. Chem. Soc.* **135**, 9267–9270 (2013)
Ni-Mo	0.05	*ACS Catal.* **3**, 166–169 (2013)
p-1T-MoS_2	0.5 (153 mV)	*J. Am. Chem. Soc.* **138**, 7965–7972 (2016)
CoP	0.046	*Angew. Chem. Int. Ed.* **53**, 5427–5430 (2014)
FeP/Ti	0.277	*ACS Nano.* **8**, 11101–11107 (2014)

Supplementary Table 6. Fitting parameter values derived from the analysis of impedance spectra recorded in 0.5 M H_2SO_4 solution at 298 K

Catalysts	R_s (Ω)	R_1 (Ω)	n_1	CPE_1	R_{ct} (Ω)	n_2	CPE_{ct}	C_{dl} (mF)
Fe/GD	3.1	1.19	0.94	3.3×10^{-4}	914.3	0.88	6.7×10^{-4}	0.63
Ni/GD	3.39	3.63	1	3.0×10^{-5}	351.7	0.70	5.8×10^{-4}	0.29
GDF	5.12	15.17	0.39	5.2×10^{-3}	1493	0.96	2.0×10^{-4}	0.19
CC	3.83	182.2	0.93	2.3×10^{-4}	2.9×10^{4}	0.96	7.3×10^{-5}	0.08

Supplementary Notes

Supplementary Note 1: XPS and Raman characterization of CC and GDF

For pure CC, the C 1s XPS spectrum shows four typical peaks assignable to C–C (284.3 eV), C–O (284.9 eV), C=O (287.9 eV), and O-C=O (290.3 eV), respectively. For GDF, high resolution XPS spectra of C 1s can be deconvoluted into four sub-peaks located at 284.4, 285.1, 286.6, and 288.2 eV, corresponding to the C 1s orbital of C–C (sp^2), C–C (sp), C–O, and C=O, respectively. The peaks of C–O and C=O can be ascribed to the absorption of air and the oxygen-containing groups on CC surface. The area ratio of the sp– and sp^2– hybridized carbon atoms was 2, confirming that the benzene rings link with others by diine in as-prepared GD structure. Compared with GDF, the C 1s XPS peaks of both Ni/GD and Fe/GD (Supplementary Fig. 2) all featured a satellite at 290.0 eV arising from the π–π^* transition, which could be due to the restoration of the conjugated structure. The area ratio of the sp- and sp^2-hybridized carbon atoms of the samples remained equal to 2, suggesting that the anchoring of Ni/Fe atoms occurred without breaking any covalent bonds. Moreover, for pure CC, Raman spectrum shows two peaks located at 1373.3 cm^{-1} and 1593.3 cm^{-1}. For GDF, Raman spectrum exhibits four characteristic peaks located at 1387.2, 1589.3, 1953.1 and 2189.8 cm^{-1}, respectively. The peak at 1387.2 cm^{-1} corresponds to the breathing vibration of sp^2 carbon domains in aromatic rings (D band). The peak located at 1589.3 cm^{-1} corresponds to the first-order scattering of the E_{2g} mode observed for in-phase stretching vibration sp^2 carbon domains in aromatic rings (G band). The intensity ratio of the D and G bands is 0.77, which indicates the high order and low defects in the GD films, and the GD films are composed of multilayers. The peaks at 2189.8 and 1953.1 cm^{-1} correspond to vibrations of the triple bonds (–C≡C–C≡C–), indicating the successful coupling reaction. Compared with GDF, the diffraction peaks of the diyne groups of Ni/GD and Fe/GD (Supplementary Fig. 2) shifted slightly, consistent

with the formation of chemical bonds after TM atoms anchoring. The ratio of the D and G band intensities for both Ni/GD (0.87) and Fe/GD (0.85) are larger than that of GDF (0.77), suggesting more defects had formed.

Supplementary Note 2. The pre-edge derivative of XANES spectra of Fe/GD and Ni/GD

As discussed in the manuscript (please see the manuscript for details), the most favorable adsorption site for single metal atoms (e.g., Fe and Ni) on monolayer graphdiyne (GD) is all in the alkyne ring. Further for intercalation of single metal atoms between GD layers, the most favorable adsorption site for the Ni/Fe atoms is A1 (Supplementary Fig. 13), we can find the Ni/Fe atoms lies between two GD layers. The case is more like that of metallocenes (e.g., ferrocene and nickelocene), a type of organometallic compound consisting of a central metal atom between two cyclopentadienyl rings[1-3]. Previous studies have provided the best understanding of the interaction between metal center and two organic unites. The metal can be regarded as in a zero oxidation state[4-5]. This means that ferrocene consists of an Fe(0) center and two cyclopentadienyl ligands.

It is a very important evidence from previous literature[6] concerning the metallocenes from which we can find the main peaks of ferrocene and nickelocene in two metallorganic compounds are mainly appeared around 7130 eV and 8344 eV, respectively. In our experimental XANES spectra (Supplementary Fig. 10), the main peaks of Fe/GD and Ni/GD are located at 7125 eV and 8340 eV, respectively. Both Fe/GD and Ni/GD show the shifts of the adsorption edge to smaller energies compared to that of ferrocene and nickelocene, respectively. Besides, there is no doubt that the zero-valence atoms, compared to the zero-valence bulk materials and clusters (single atoms), the interaction may be more complex from that of their own due to their atoms level size. And confinement of electrons leads to a discrete energy level distributions and a distinctive HOMO-LUMO gap. These lead to quantum size and small size effects, resulting in the more change of XANES spectrum. According to the above-discussed, it can be considered that Fe/GD (or Ni/GD) consists of an Fe(0) [or Ni(0)] center.

Supplementary References

1. Kealy, T. J. & Pauson, P. L. A new type of organo-iron compound. *Nature* **168**, 1039–1040 (1951).
2. Wilkinson, G., Rosenblum, M., Whiting, M. C. & Woodward, R. B. The structure of iron bis-cyclopentadienyl. *J. Am. Chem. Soc.* **74**, 2125–2126 (1952).
3. Dunitz, J. D. & Orgel, L. E. Bis-cyclopentadienyl–a molecular sandwich. *Nature* **171**, 121–122 (1953).
4. Bell, C. F. Syntheses and physical studies of inorganic compounds. Pergamon: New York, 1972, Chapter 20.
5. Strohfeldt, K. A. Essentials of inorganic chemistry: for students of pharmacy, pharmaceutical sciences and medicinal chemistry. John Wiley & Sons, Ltd. 2015, Chapter 8.
6. Ruiz-Lopez, M. F., Loos, M., Goulon, J., Benfatto, M. & Natoli, C. R. Reinvestigation of the EXAFS and xanes spectra of ferrocene and nickelocene in the framework of the multiple scattering theory. *Chem. Phys.* **121**, 419–437 (**1988**).
